# The Protective Effect of Quercetin on Hydrogen Peroxide-Induced Oxidative Damage in Caco-2 Cells Is Enhanced by Its Loading in Mesoporous Silica Nanoparticles

**DOI:** 10.3390/pharmaceutics18030316

**Published:** 2026-03-01

**Authors:** Alexis Matadamas-Ortiz, Prospero Di Pierro, Angela Sorrentino, Ivana Caputo, Gaetana Paolella, Antonio Montefusco, Carlos Regalado-González

**Affiliations:** 1 Departamento de Investigación y Posgrado en Alimentos, Facultad de Química, Universidad Autónoma de Querétaro, C.U., Cerro de las Campanas s/n, Col. Las Campanas, Querétaro 76010, Mexico; amatadamas717@alumnos.uaq.mx; 2 Department of Agricultural Sciences, University of Naples Federico II, Via Università 100, 80055 Portici, Italy; prospero.dipierro@unina.it; 3 Centre for Innovation and Development in the Food Industry (CAISIAL), University of Naples Federico II, Via Università 133, 80055 Portici, Italy; angela.sorrentino@unina.it; 4 Department of Chemistry and Biology, University of Salerno, 84084 Fisciano, Italy; icaputo@unisa.it (I.C.); gpaolella@unisa.it (G.P.); amontefusco@unisa.it (A.M.)

**Keywords:** quercetin, mesoporous silica nanoparticles, oxidative stress, Caco-2 cells

## Abstract

**Background:** Quercetin (Q) can reduce cellular oxidative stress, though it is susceptible to degradation in physiological conditions. Through adsorption and protection of Q, mesoporous silica nanoparticles (MSNs) could enhance its bioactivity. This work aimed to determine the effect of Q loading in MSN and in its aminated (A-MSN), carboxylated (C-MSN) or thiolated (T-MSN) derivatives on its Caco-2-cytoprotective effect against H_2_O_2_-induced oxidative stress. **Methods:** The mesoporous silica materials were characterized (FT-IR, ζ-potential, TGA), and their cytotoxicity was assessed; then, they were loaded with Q and incubated with Caco-2 cells prior to oxidative stress induction, and the cytoprotective effect was evaluated through measurement of cell viability. **Results:** None of the nanoparticles showed toxicity to Caco-2 cells. A-MSN showed the highest Q loading capacity (5.26% ± 0.06%), due to hydrogen-bonding interactions. C-MSN clearly enhanced the Q cellular uptake compared to the other nanoparticles. Oxidative stress decreased Caco-2 cell viability, which was prevented by 100 µM free Q after 18 h incubation. In contrast, higher cell viability than in non-stressed cells was observed with the same Q concentration loaded across all nanoparticle types. **Conclusions:** Despite the high instability of free quercetin under cell culture conditions, it exerted a time-dependent cytoprotective effect against H_2_O_2_-induced oxidative stress that was enhanced upon loading into nanoparticles. Prior release of the Q molecule in the medium is ineffective, and the presence of the loaded material is required.

## 1. Introduction

Mesoporous silica nanoparticles (MSNs) as carriers have good properties, such as large pore volume (up to 2.5 cm^3^/g), high surface area (800–1000 m^2^/g), modifiable pore diameter (2–50 nm), functionalizability, biodegradability, high chemical and biological stability, and good biocompatibility [1,2]. MSNs could deliver therapeutic molecules with poor membrane permeability that can be adsorbed onto this material via its surface silanol groups (Si-OH) [3]. Moreover, the ease with which MSNs can be modified by adding ionic or covalent ligands allows control over loading capacity and the release of molecules in different media [4].

Quercetin (2-(3,4-hydroxyphenyl)-3,5,7-trihydroxy-4H-chromen-4-one) is a flavanol found as an aglycone or in conjugated derivative form [5]. The Q structure possesses two aromatic rings connected by a three-carbon γ-pyrone ring. Its antioxidant property is due to the catechol group present in the aromatic ring (B) and to -OH groups in the aromatic ring (A) [6]. Q exhibits strong free radical scavenging activity and can neutralize reactive oxygen species (ROS) by donating electrons or hydrogen atoms, thereby reducing cellular oxidative stress [7].

Oxidative stress in cells refers to the disruption of the balance between ROS generation and the cells’ capacity to neutralize and remove them. This process contributes to the development of epithelial damage and gastrointestinal diseases, such as intestinal barrier dysfunction [8,9]. H_2_O_2_ is one of the main ROS in cells and is key to numerous physiological processes. H_2_O_2_ is poorly reactive; however, it can generate reactive hydroxyl radicals upon exposure to UV light or transition metal ions (e.g., iron), thereby generating oxidative stress [10].

The small intestinal epithelium is a physical barrier against the entry of toxins, bacteria, and other harmful substances into the blood [11]. It has been indicated that protecting the intestinal barrier from H_2_O_2_ and interleukin-6-induced damage may be a practical approach to preventing intestinal inflammation [12].

Q has shown antioxidant effects against H_2_O_2_-induced oxidative damage; however, its poor solubility, which at body temperature results in a crystalline form, limits its bioaccessibility and bioavailability [13]. Additionally, information on the bioactivity of polyphenols (including flavonoids) has been obtained from in vitro cell culture experiments, conditions in which these compounds have been reported to be highly unstable [14]. There is a lack of clinical trials validating quercetin’s effectiveness due to its high instability in physiological systems, leading to degradation within 2 h [15]. This disadvantage could be overcome by loading quercetin into mesoporous materials, which can enhance its effects by protecting it from degradation under cell culture conditions, promoting controlled release, and increasing solubility and cellular uptake [16]. These properties are directly linked to the nanoparticles’ surface chemistry that regulates their behavior in physiological solutions. The loading efficiency and the release kinetics in different media are determined by the interaction strength between the mesoporous surface and Q. Furthermore, the material’s surface chemistry promotes interactions between the materials and the cell membrane. According to Siddiqui et al. [17], factors affecting cellular uptake include surface charge and particle size (also influenced by surface charge). Therefore, it can be assumed that the surface chemistry of the mesoporous silica used as a vehicle also affects the efficiency of Q’s cytoprotective effect in the cell culture medium. However, there are few experiments evaluating this effect.

Because of their homology with intestinal epithelial cells, Caco-2 cells have been endorsed by the US Food and Drug Administration (FDA) as an in vitro model for human intestinal absorption and metabolism [18]. This work aimed to elucidate the effect of Q loading into different types of functionalized mesoporous silica nanoparticles on its cytoprotective activity against H_2_O_2_-induced cell damage in a Caco-2 intestinal cell model.

The novelty of this research lies in the information on the cellular effects of quercetin delivered via mesoporous silica materials with different surface functionalizations. This helps clarify how the surface chemistry of mesoporous vehicles affects the bioactivity of loaded quercetin by altering the kinetics of release, cellular uptake, and interactions with the tested cells.

## 2. Materials and Methods

### 2.1. Materials

The ethanol (>99%), hexadecyltrimethylammonium bromide (CTAB), tetraethyl orthosilicate (TEOS), amine propyltrimethoxysilane (APTES), mercaptopropyltrimethoxysilane (MPTMS), succinic anhydride (SA), chloroform, NaOH, CaCl_2_, quercetin hydrate (>95%), dimethyl sulfoxide (DMSO), pepsin, pig biliary salts, and pancreatin were purchased from Sigma-Aldrich (St. Louis, MO, USA). The 3-(4,5-dimethylthiazol-2-yl)-2,5-diphenyltetrazolium bromide (MTT), minimum essential medium (MEM), fetal bovine serum (FBS), penicillin, streptomycin, and Hanks’ balanced salt buffer solution (HBSS) were purchased from Gibco (Gaithersburg, MD, USA). Water, acetic acid, methanol, and acetonitrile were HPLC grade and were acquired from Carlo Erba Reagents srl (Cornaredo, Milan, Italy).

### 2.2. Cell Cultures

Caco-2 cells (human colorectal adenocarcinoma cell line) were used as an intestinal cell model. The cell line was acquired from Interlab Cell Line Collection (Instituto Nazionale per la Ricerca sul Cancro, Genova, Italy). For culture, MEM was used, supplemented with FBS (10% *v*/*v*), L-glutamine (0.2 mM), penicillin (100 units/mL), and streptomycin (0.1 mg/mL). Cells were incubated at 37 °C in a humidified incubator (with 5% CO_2_ added) in 75 cm^2^ culture flasks. Confluent cell monolayers (50–70%) were used for different assays [19]. For all reported experiments, Caco-2 cells were used at passages 29–35.

### 2.3. Synthesis and Characterization of Mesoporous Silica Nanoparticles (MSNs)

#### 2.3.1. Synthesis and Functionalization

Synthesis of Mobil Composition of Matter No. 41 (MCM-41) type MSN was carried out following the method of Kresge et al. [20], employing CTAB as pore-directing agent and TEOS as silica source. CTAB (0.5 g) was dissolved in distilled water (240 mL), and 2 N NaOH (1.75 mL) was added. The mixture was heated to 80 °C, and then TEOS (2.5 mL) was added dropwise. After 2 h of reaction, the resultant powder was recovered by filtration using a sintered glass filter (4–5.5 μm of porosity) and washed twice with distilled water and twice with absolute ethanol. Surfactant removal was achieved by calcination at 500 °C for 5 h.

Amine-functionalization of mesoporous silica has proven to enhance the interaction with quercetin in physiological pH conditions by the formation of hydrogen bonds and even electrostatic interactions (between protonated amine groups and deprotonated -OH in Q structure). On the contrary, carboxyl groups on the surface of mesoporous silica exert repulsive forces between deprotonated -OH groups of quercetin and carboxylate ions. In physiological conditions, thiol groups are neutral, and the possible hydrogen bonds formed with hydroxyl groups of quercetin are weak due to the low electronegativity of sulfur. These groups were used as models to achieve attractive, repulsive, and neutral interactions between the quercetin and the mesoporous material.

To obtain aminated MSN (A-MSN), carboxylated MSN (C-MSN), and thiolated MSN (T-MSN), the methodology described in a previous work was used [16]. To obtain A-MSN, MSN (100 mg) was suspended in 20 mL of ethanol, and 1 mL of APTES was added. This suspension was stirred for 24 h at room temperature, and the solid material was recovered by filtration and washed twice with ethanol. For T-MSN, this methodology was applied, replacing APTES with MPTMS. For C-MSN, the same amount of MSN was suspended in a 2% (p/v) succinic anhydride solution (50 mL) in chloroform. After filtration, the mixture was washed sequentially with chloroform, deionized water, and ethanol. In a previous study, mesoporous silica materials were characterized by particle size, polydispersity index, and ζ-potential. Furthermore, the mesoporous structure of MSN was confirmed by gas physisorption analysis, and the textural parameters were similar to those reported by other authors [16].

#### 2.3.2. Quercetin (Q) Loading

To maximize quercetin loading in the material, a dispersion was prepared by suspending 100 mg of particles in 50 mL of a Q-saturated ethanol solution (2 mg/mL). The dispersion was stirred (150 rpm) for 24 h at room temperature to equilibrate Q concentrations between the nanoparticles and the solution, followed by total solvent volatilization under vacuum. The solvent was chosen to avoid toxicity concerns. Then, the particles were re-suspended quickly in 50 mL of ethanol, vortexed for 1 min, and finally centrifuged at 2200× *g* for 15 min. The pellet was recovered and dried at room temperature. Particles loaded with Q were represented with “/Q” next to the name of the particle.

#### 2.3.3. Thermogravimetric Analysis (TGA)

The presence of the -NH_2_, -COOH, and -SH groups in the functionalized materials, as well as the amount of Q loaded in the particles, was investigated by thermogravimetric analysis (TGA) using a differential thermal analysis instrument (TGA 7/DZ, Perkin Elmer, Kanagawa, Japan) with nitrogen as the carrier gas. The particles were weighed (3 mg–6 mg) and heated up to 600 °C, at a rate of 10 °C/min. To determine the existence of different functional groups in the studied silica materials, the weight losses of MSNs and their derivatives were compared. To calculate the loading capacity, the weight loss of loaded and unloaded particles was compared. This procedure was performed in triplicate. The loading capacity was used in the following experiments to normalize the nanoparticle dose in terms of quercetin content.

#### 2.3.4. FT-IR Spectroscopy

To investigate the interaction between Q and the different matrices, FT-IR spectra (4000–650 cm^−1^) of the empty or Q-loaded MSNs were obtained by using a Horiba IR system (Mod. Jobin Yvon LabRAM IR2, Kyoto, Japan). Each spectrum was obtained once, averaged from 8 scans at a resolution of 4 cm^−1^.

#### 2.3.5. ζ Potential Measurement

To investigate the attraction/repulsion forces between particles in the cell culture media, they were suspended (0.1 mg/mL) in supplemented MEM, and the ζ potential was calculated through electrophoretic measurements of three independent samples utilizing a ZetaSizer Nano Instrument (Malvern Instruments, Worcestershire, UK).

### 2.4. Cytotoxicity Assay

To determine the effects of different particle types on Caco-2 cell viability, the MTT assay was executed as described by Paolella et al. [19]. In 96-well microplates, Caco-2 cells were seeded at 7 × 10^3^ cells/well and incubated in a 5% CO_2_ atmosphere at 37 °C. Then, the MSN, A-MSN, C-MSN, or T-MSN was added at 10, 100, and 500 µg/mL. At the same time, Q solutions in DMSO (0.1% *v*/*v*) were added at concentrations of 25, 100, and 200 µM. After 24 h of incubation with the particles, the cells were incubated for 1 h with an MTT salt solution. After medium removal, the formed formazan crystals were dissolved in 100 µL of DMSO by oscillatory stirring. In a microplate reader spectrophotometer (SpectraMax MiniMax, Molecular Devices, Silicon Valley, CA, USA), the absorbance of the samples and the background were measured at 595 nm and 655 nm, respectively. Cell viability relative to blank, also called relative viability (RV), was calculated by using Equation (1):

RV (%) = (sample − background)/(blank − background) × 100(1)

### 2.5. Quercetin (Q) Release Test

An accurate amount of each particle type was dispersed in a fixed volume of MEM to obtain a concentration of 3 mg/mL. Then, in 96-well microplates, the suspensions were diluted with MEM to obtain Q concentrations of 25, 50, and 100 µM in a total volume of 300 µL. A solution of 100 µM of free Q was used as a control. To determine the kinetics of Q release, separate wells were used for each time interval (1, 2, 4, 8, 24, and 48 h). The microplates were placed in the dark at 37 °C ± 2 °C, without stirring. At fixed time intervals, the total volume of each well was measured, and the mixture was centrifuged at 16,000× *g* for 10 min. Then, the supernatant was recovered and mixed with an extraction solution (1:7, *v*/*v*) composed of ethyl acetate and ethanol (9:1, *v*/*v*). The mixture was vortexed for 10 s and centrifuged at 16,000× *g* for 10 min, and the supernatant was dried in a fume extraction cabinet at room temperature. Finally, the residue was dissolved in the mobile phase solvent (100 µL) to measure the Q concentration by HPLC. A Jasco PU-4180 RHPLC system equipped with an MD-4010 photo diode array detector (Jasco International, 11–10, Myojin-cho 1-chome, Hachioji, Tokyo, Japan) and a C18 SunFire column (4.6 × 150 mm, 5.0 µm, Waters, Milford, MA, USA) was used. The mobile phase consisted of 45% aqueous solution of 0.1% (*v*/*v*) acetic acid (WAc), 40% (*v*/*v*) methanol (Met), and 15% (*v*/*v*) acetonitrile (ACN) at a flow rate of 0.5 mL/min, with a column temperature of 30 °C. Detection was carried out at 370 nm (Q).

### 2.6. Cellular Uptake

Caco-2 cells were seeded in 6-well plates (2 × 10^4^ cells/well) and cultured for 15 days, with medium changes every 48 h (week 1) or 24 h (week 2). Prior to treatment, monolayers were washed with HBSS and preincubated at 37 °C for 30–60 min. Then, the cells were incubated for 60 min with different MSN formulations (equivalent to 50 µM quercetin). After incubation, the medium and two washes were collected using specific buffers to maintain the nanoparticles’ neutral charge: HBSS (MSN), buffer pH 8.0 for A-MSN, and buffer pH 5.3 for C-MSN. For T-MSN, HBSS was supplemented with L-cysteine to trigger disulfide bond cleavage. Cells were detached with 20 µM trypsin (5 min), neutralized with medium, and centrifuged at 780× *g* for 5 min. Pellets were washed using the respective buffers and stored at −20 °C. Pellets were resuspended in 0.1 M phosphate buffer with 1% Triton X-100, vortexed, and sonicated (15 min, 300 W) for lysis. After protein quantification via Bradford assay, quercetin (Q) was extracted using ethyl acetate:ethanol (9:1 *v*/*v*), vortexed, and centrifuged (16,000× *g*, 10 min). The supernatant was dried at 30 °C, and the residue was redissolved in 100 µL of WAc:Met:ACN (45:40:15 *v*/*v*). Finally, Q concentration was determined by HPLC (20 µL injection).

### 2.7. Effect on H_2_O_2_-Induced Oxidative Stress

Before evaluating the effects of treatments on H_2_O_2_-induced cell damage, an appropriate level of cellular oxidative stress was established, as a high survival rate leads to insignificant inductions. At the same time, excessive H_2_O_2_ can cause irreversible cell damage [21]. Despite the H_2_O_2_ concentration being the same across all independent experiments, cell damage showed slight differences. Thus, to obtain comparable values among independent experiments, Equation (2) was applied to calculate RV recovery, defined as the percentage of RV lost due to the effect of H_2_O_2_ that was recovered by the treatment with Q:

RV recovery (%) = (RV_Treated_ − RV_Stressed_)/(100 − RV_Stressed_) (2)

where:

RV_Treated_: RV of cells exposed to Q (loaded in particles or not) and to H_2_O_2_-induced stress.

RV_Stressed_: RV of cells exposed to H_2_O_2_-induced stress.

In biological terms, RV recovery normalizes the cytoprotective effect based on the observed cell damage in independent experiments.

#### 2.7.1. Effect of Loaded Nanoparticles

The effect of particles loaded with Q on Caco-2 cells under H_2_O_2_ stress was investigated. Cells were incubated in 96-well plates for 18 h in supplemented MEM containing different Q-loaded particles at equivalent Q concentrations of 12.5, 25, 50, or 100 µM. The same concentrations of free Q were tested at two incubation times (2 and 18 h). These tests were followed by the addition of diluted H_2_O_2_ in PBS to a final concentration of 400 µM in each well. Cell incubation was performed for 1 h, followed by the MTT assay as previously described, with the exposure time extended by 1 h. Considering this, exposure time to H_2_O_2_ was 2 h [22].

#### 2.7.2. Effect of the Loaded Nanoparticles’ Release Medium

To address the effect of Q released from loaded particles into the culture medium, suspensions of each mesoporous silica type were prepared by adding the particles to supplemented MEM at Q concentrations of 25, 50, and 100 µM. After 24 h of incubation at 37 °C, the suspensions were centrifuged (780× *g* for 5 min) to remove the particles. The cell culture medium recovered (supernatant) was used to seed Caco-2 cells for 18 h at 37 °C, followed by the addition of H_2_O_2_ and cell viability evaluation, as depicted in Section 2.7.1.

### 2.8. Statistical Analysis

All experiments were conducted in triplicate, and the results were reported as mean ± standard deviation (SD). An analysis of variance (ANOVA) was applied where appropriate, and to determine significant differences between treatments or between treatments and the control; the Tukey’s or Dunnett’s tests (*p* < 0.05) were used, respectively.

## 3. Results and Discussion

### 3.1. Thermogravimetric Analysis

**Figure A1:**
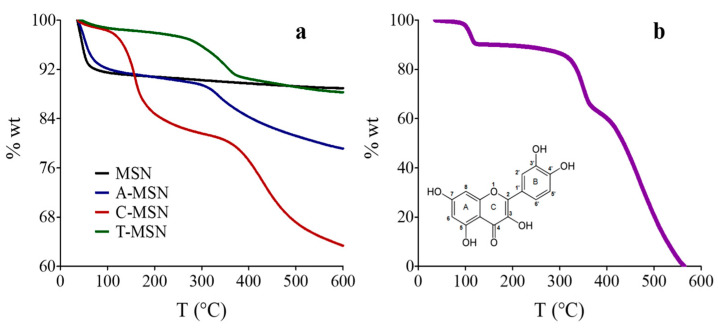
TGA curves of (**a**) MSN, A-MSN, C-MSN, and T-MSN, and (**b**) quercetin. The mass loss of functionalized mesoporous silica in the 250–450 °C range is associated with the degradation of 3-mercaptopropyl [65], 3-aminopropyl [28], and carboxyl [30] groups, indicating the success of the functionalization process. The mass loss of quercetin starting at 300 °C is related to C-ring breakage or the loss of the A or B rings [66].

**Figure A2:**
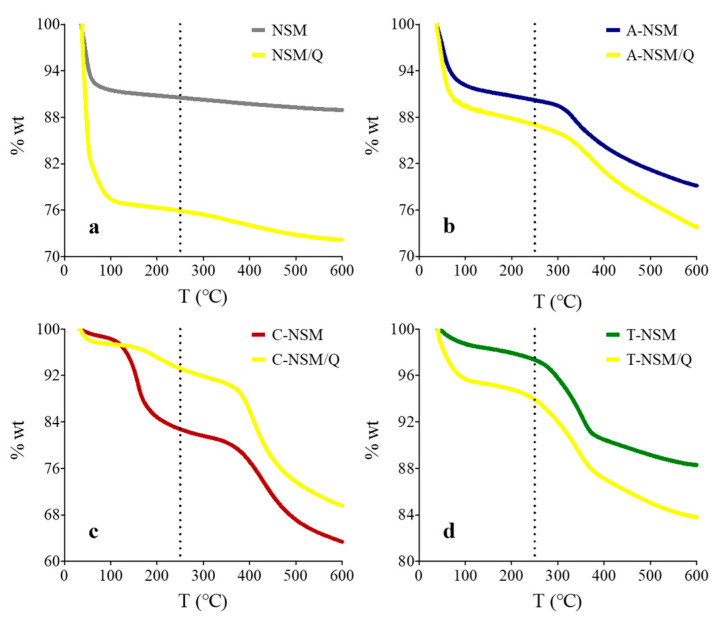
TGA curve comparison of empty and Q-loaded (**a**) MSN, (**b**) A-MSN, (**c**) C-MSN, and (**d**) T-MSN. To de-termine the loading capacity of each material, sample weights were normalized to 100% at 250 °C (verti-cal dashed line). It was conducted for calculation purposes and is not shown in the plot. This procedure was used to exclude initial mass loss due to dehydration and residual solvents removed.

TGA results confirmed the materials’ functionalization (Figure A1). The loading capacity of the particles was evaluated by measuring the weight loss difference between loaded and unloaded samples (Figure A2). The loading capacity of each kind of particle is reported in Table 1. Amine functionalization significantly increased the particle loading capacity, possibly by increasing the number of hydrogen bonds between Q and MSN, facilitated by the presence of amino groups. The C-MSN and T-MSN showed a lower capacity to accommodate the Q molecule, which is related to the decrease in pore volume and size due to the addition of functional groups on the surface of MSNs. Regarding the different functionalities of the MSNs, all nanoparticles showed high negative ζ potential (Table 1), with little difference. These results agree with a previous report indicating that the bio-reactivity of nanoparticles is affected by the adsorption of biological molecules, forming a protein corona that depends on surface chemistry [23].

### 3.2. FT-IR Spectroscopy

**Figure 1:**
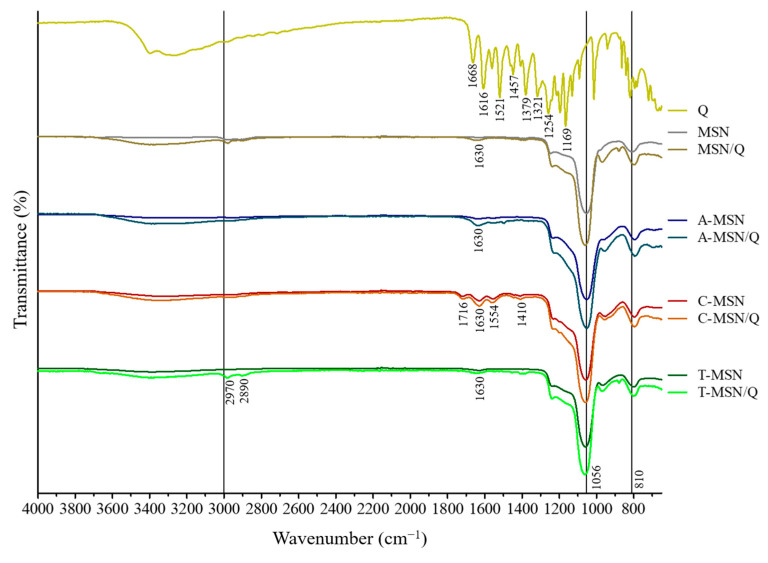
FT-IR spectra of quercetin and bare or Q-loaded mesoporous silica materials.

FT-IR spectroscopy results are shown in Figure 1. The FT-IR spectrum of quercetin is well described, and the bands occurring due to vibration of the molecule’s bonds are observed at 1668 cm^−1^ (aryl ketonic C=O stretching), 1616 cm^−1^ (aromatic ring C=O stretching), 1521 cm^−1^ and 1457 cm^−1^ (aromatic ring C=C stretching), 1379 cm^−1^ (phenol O–H bending), 1321 cm^−1^ (in-plane aromatic C–H bending), 1254 cm^−1^ (aryl ether ring C–O stretching), and 1169 cm^−1^ (hydroxyl O–H bending or methoxy C–O stretching) [24,25,26].

For MSN and its functionalized derivatives, the typical bands of silica materials corresponding to asymmetric and symmetric stretching of the Si–O bond (1056 cm^−1^ and 810 cm^−1^, respectively) are visible [27]. The functional groups of A-MSN were confirmed by the observation of subtle bands at 1630 cm^−1^ and 1560 cm^−1^, consistent with the stretching and bending of the N–H bond [28,29]. The band at 1630 cm^−1^ was similarly observed in other functionalized mesoporous materials and is attributed to the vibration of –OH groups of water adsorbed within their nanochannels. Similarly, the carboxyl groups of C-MSN were confirmed by bands corresponding to the C=O stretching of the carboxylic group (1716 cm^−1^) [30], and the symmetric and asymmetric stretching of the carboxylate anion (1554 cm^−1^ and 1410 cm^−1^, respectively) [29]. A faint band in the 2600–2540 cm^−1^ range, typically assigned to S–H stretching [31], was not detected in FT-IR spectra of the T-MSN samples. Nevertheless, the presence of the –SH group was confirmed by thermogravimetric analysis (Section 3.1). Upon loading the mesoporous materials with quercetin, a significant decrease in transmittance was observed between 1700 and 650 cm^−1^, a region corresponding to the dominant bands of pure quercetin. This spectral overlapping confirms the successful incorporation of quercetin into the mesoporous structure. Furthermore, the observed changes in intensity were inversely correlated with quercetin content, consistent with the thermogravimetric data. Notably, the most pronounced decrease in the transmittance was recorded for the A-MSN sample, which exhibited the highest quercetin loading (see Section 3.1). Additionally, the breadth and intensity of the 3700–3000 cm^−1^ bands (related to –OH vibration) of all materials were enhanced by the addition of Q, which suggests its association with the silica matrix through H-bonds.

### 3.3. Mesoporous Silica Nanoparticle Cytotoxicity

The cytotoxicity of mesoporous silica particles is a particle size-, administered dose-, and cell type-dependent process that involves ROS production and alterations in membrane integrity generated by cellular uptake [32].

**Figure 2:**
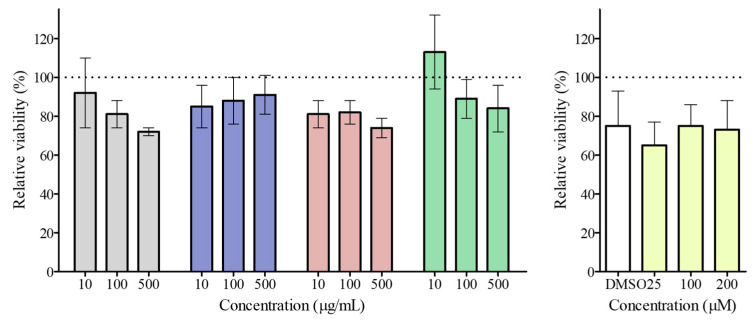
Relative viability of Caco-2 cells after 24 h exposure to MSN (grey), A-MSN (blue), C-MSN (red), T-MSN (green), and quercetin (yellow). Data shown are the means ± SD (*n* = 3).

The results of the MTT assay (Figure 2) revealed stable Caco-2 cell viability after 24 h, regardless of particle concentration, up to 500 µg/mL (*p* < 0.05).

Earlier studies have documented similar results using this cell line. Amine-functionalized MSN at 10 µg/mL to 500 µg/mL did not exhibit cytotoxicity after 24 h of exposure [33]. After 72 h, a cytotoxic effect of particles was observed at 500 µg/mL (60.6% viability). This may be due to reduced nutrients in the medium, which halted cell proliferation. In addition, MSN functionalized with groups that confer positive, negative, or neutral electrical character (-NH_2_, -PO_3,_ and -CH_3_) showed no cytotoxicity up to 500 µg/mL [34].

Furthermore, up to a Q concentration of 200 µM, no toxic effect was observed after 24 h (*p* < 0.05). This differs from existing evidence of apparent viability inhibition in Caco-2 cells exposed to Q concentrations > 20 µM, which is associated with increased intracellular ROS [35]. However, other works reported that Q showed a faint cytotoxic effect at very high concentrations [36,37]. Thus, it is clear that different experimental conditions may affect the biological activity of Q or, more generally, of natural compounds. In contrast, the beneficial effects associated with flavonoids are attributed to their direct antioxidant activity, which allows them to sequester ROS and reactive nitrogen species (RNS) via the arrangement of the hydroxyl groups on their B ring, which can donate hydrogen and electrons [38].

### 3.4. Kinetics of Quercetin Release

For all samples and incubation times, the amount of Q released was below the detection limit (15 µM); therefore, it was not possible to make a precise estimate relative to the theoretical initial value. However, this result indicates a very low concentration of Q in the culture medium. Minimum essential medium (MEM) composition includes salts, amino acids, vitamins, glucose, sodium pyruvate, and phenol red. There is a lack of information about flavonoids’ stability in MEM; however, several studies have been conducted on flavonoids’ stability in Dulbecco’s modified Eagle medium (DMEM), which reveals a similar composition, containing higher amino acid concentration (twice) and a higher vitamin dose (4 times) [39]. It has been shown that Q is not stable under cell culture conditions, such as those provided by DMEM. Hu et al. [40] observed that Q in DMEM at pH 7 and 8 decreased significantly (nearly 50%) within 1 h and then decreased gradually over the next 5 h. These authors also evaluated the individual effects of the medium supplements (HEPES, sodium bicarbonate, glucose, sodium pyruvate, penicillin, streptomycin, and FBS), where only NaHCO_3_ significantly affected the stability of this compound. Xiao & Högger [41] reported that Q incubated in DMEM (10 µM) in the absence of cells exhibited a half-degradation time of 17.96 min; however, it should be noted that the degraded or metabolized polyphenol may display different bioactivity from that of the original compound. Cao et al. [42] conducted a UPLC-MS-MS analysis to assess the stability of Q in DMEM at 37 °C; after 180 min of incubation at 50 µM, Q was hardly detectable. The degradation of Q in the medium was attributed to the creation of a redox system by the DMEM constituents or to the creation of Q complexes with divalent cations by chelation. On the other hand, flavonoids, including Q, can form complexes with plasma proteins such as bovine serum albumin (BSA), suggesting that Q–protein complexes can be removed by centrifugation during sample processing [43,44,45]. In this regard, based on these reports, we assumed that quercetin is highly unstable in the MEM culture medium.

### 3.5. Cellular Uptake

**Figure 3:**
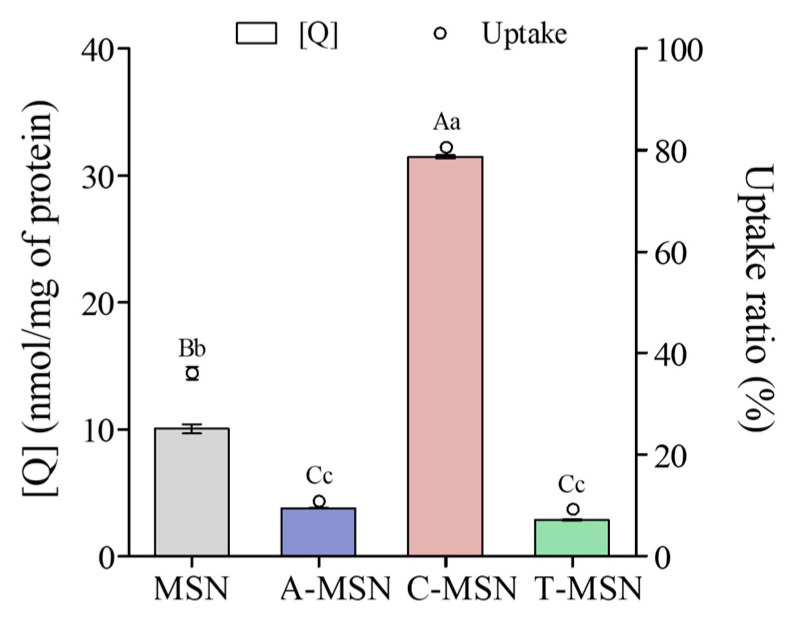
Caco-2 cells’ quercetin cellular uptake. Data shown are the means ± SD (*n* = 3). Columns indicating quercetin concentration not showing the same uppercase letter are significantly different (*p* < 0.05). Circles indicating the uptake ratio of quercetin not displaying the same lowercase letter are significantly different (*p* < 0.05).

Functionalized mesoporous silica increases drug absorption through an increase in drug solubility and cellular internalization [46]. Figure 3 shows the Q cellular uptake by the Caco-2 cells. As shown clearly, C-MSN exhibited the highest uptake ratio (80.48 ± 0.37% of the particles’ initial Q content). On the contrary, cells internalized approximately only 10% of the Q loaded in the A-MSN and T-MSN, which is lower than the 36.03 ± 1.28% internalized from MSN.

Particle size is among the most critical factors affecting the delivery of MSN-entrapped agents to the target site and is affected by pH, among other factors. In this regard, smaller particles are usually preferred due to their superior cellular uptake [17]. Endocytosis routes for nanoparticles are tightly related to particle size. The phagocytosis pathway is used to internalize particles > 0.5 μm, limited to macrophages, dendritic cells, and neutrophils [47]. Nanoparticles with a diameter > 200 nm trigger the complement system and are rapidly eliminated from the bloodstream by the mononuclear phagocytes and the reticuloendothelial system [48]. On the other hand, pinocytosis takes place in all cell types [49]. Particles < 200 nm are internalized via the clathrin-mediated and caveolae-mediated pathways [3,50,51]. These endocytosis pathways transport the nanoparticles previously coated by plasma proteins present in physiological solutions [47]. In a previous work, we reported that MSN/Q, A-MSN/Q, and T-MSN/Q formed aggregates with particle sizes in the micron scale when suspended in PBS at pH 7.4 (similar to cell culture medium). In contrast, C-MSN/Q showed a particle size of 210 nm due to strong particle repulsion [16]. Thus, the improvement in Q delivery by C-MSN could be attributed to a more uniform distribution in the cell culture medium, resulting in particles with a size that allows internalization via pinocytosis pathways.

### 3.6. Cytoprotective Effect of Quercetin (Q)

**Figure 4:**
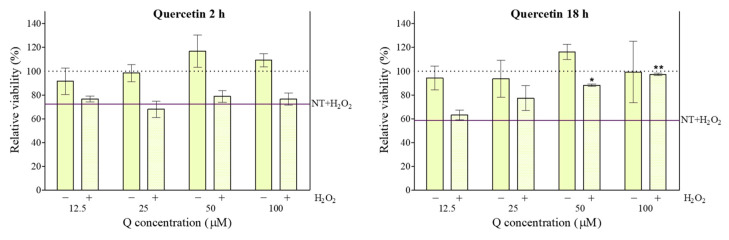
Relative viability of Caco-2 cells after 2 h or 18 h exposure to quercetin and with (+) or without (−) 2 h exposure to H_2_O_2_. Data shown are the means ± SD (*n* = 3). Treatments with H_2_O_2_ different from the control (NT + H_2_O_2_) are marked with * (*p* < 0.05) or ** (*p* < 0.01).

Free Q showed a protective effect against H_2_O_2_-induced damage, as it inhibited the reduction in cell viability at 50 µM and 100 µM (Figure 4). Even if the MTT assay gave evidence about a cytoprotective effect of Q, it only indirectly represented a demonstration of the Q antioxidant ability towards H_2_O_2_. The viability of PC-12 cells exposed to the same type of stress was protected by Q, and this was associated with reduced H_2_O_2_-induced malondialdehyde (MDA) expression and increased expression of superoxide dismutase (SOD) and glutathione (GSH) [22]. Other reports confirm Q’s protective effect against H_2_O_2_-induced oxidative damage in the Caco-2 cell model. It was observed that the reversal of cellular damage caused by H_2_O_2_ was related to the increased cell proliferation and intracellular GSH concentration, driven by up-regulation of transcription of the catalytic subunit of glutamate-cysteine ligase (GCLC) [52]. On the other hand, in a study using a gastric mucosal epithelial cell line, Q was shown to inhibit oxidative stress-induced damage by enhancing mitochondrial and mucosal barrier function and preventing inflammation by regulation of the PI3K/AKT signaling pathway [53]. The protection offered by Q against mitochondrial dysfunction in Caco-2 cells was previously associated with its capacity for cellular internalization and mitochondrial accumulation [54]. Similarly, it has been proposed that a stress response occurs in intestinal cells (Caco-2) with mitochondrial dysfunction, as evidenced by an increase in intracellular Q, partially localized to stressed mitochondria [55].

As shown, this protective effect is time-dependent: after 2 h of incubation, Q did not show protection at any tested concentration. There are some possible explanations for the time-dependent effect of Q. Its antioxidant properties are related to two mechanisms: (1) the direct capacity of Q to scavenge ROS, and (2) the indirect up-regulation of gene expression by Q, for antioxidant substances synthesis [56]. In this respect, it has been demonstrated that Q showed both pro-oxidant and antioxidant effects in jejunum cells (IPEC-J2) stressed by lipopolysaccharide (LPS), as its treatment increased extracellular H_2_O_2_ but, overall, decreased ROS production [57].

Several studies have found that oxidation of Q produces metabolites with antioxidant capacity equal to or even higher than that of the non-oxidized flavonoid, thereby maintaining its ROS-scavenging capacity [56,58]. At the same time, some Q oxidation metabolites are not pro-oxidants in themselves; however, at high concentrations, when cells are exposed to a severe oxidative environment, they can undergo chemical changes, producing pro-oxidant species. However, low concentrations of these metabolites can induce a restorative cellular antioxidant response through various signaling pathways. In this context, Q is well known for upregulating cellular antioxidant capacity via activation of Nrf2, a regulatory factor that binds to Keap1 in the cytoplasm [59]. Oxidative stress leads to conformational changes in Keap1, triggering the dissociation and activation of Nrf2, which activates antioxidant response elements [60].

**Figure 5:**
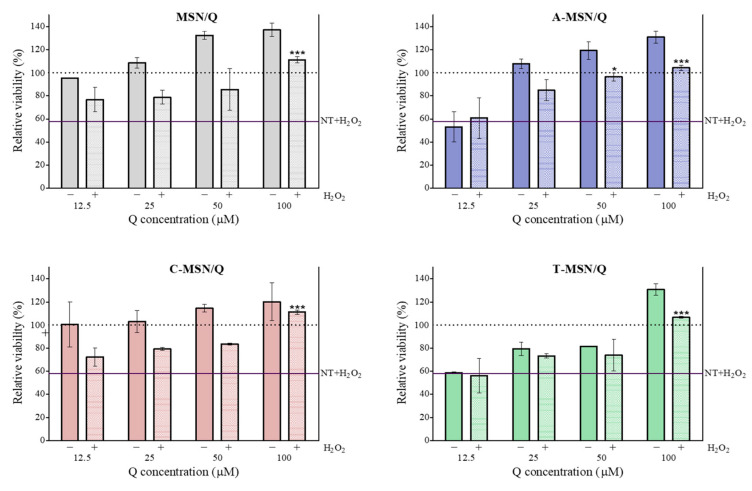
Relative viability of Caco-2 cells after 18 h exposure to F-NSM/Q and with (+) or without (−) 2 h exposure to H_2_O_2_. Treatments with H_2_O_2_ different than the control (NT + H_2_O_2_) are marked with * (*p* < 0.05) or *** (*p* < 0.001). Data shown are the means ± SD (*n* = 3).

Q at 100 µM, loaded into MSNs, retained its protective effect (Figure 5). For A-MSN, the concentration of Q needed to achieve complete inhibition of cell viability was 50 µM. Interestingly, the MSN/Q, A-MSN/Q, and T-MSN/Q showed increased cell viability in non-stressed cells at a 100 µM Q concentration (*p* < 0.05). This could be related to the cell-proliferative nature of MSNs, having potential for tissue engineering due to their inherent osteogenic and pro-angiogenic properties [61,62]. Furthermore, this effect could be due to quercetin’s beneficial activity in various cell lines, including Caco-2 cells [63], combined with the mesoporous particles’ ability to preserve and potentially enhance quercetin’s biological activity compared with untreated cells.

**Figure 6:**
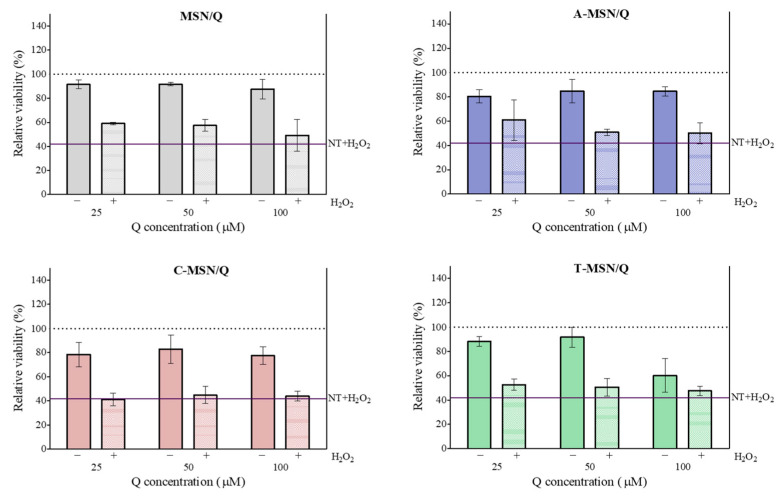
Relative viability of Caco-2 cells after 18 h exposure to F-NSM/Q release medium with (+) or without (−) 2 h exposure to H_2_O_2_. Data shown are the means ± S.D. (*n* = 3).

In contrast, when the particles were removed from the medium before its addition to the Caco-2 cells, not only was the increase in cell viability in non-treated cells abolished, but also the protective effect on stressed cells (Figure 6).

This observation agrees with the results in Section 3.3, which showed that this compound is unstable in cell culture media. It implies that the cytoprotective effect of loaded particles is not due to Q release, but we speculate that the cytoprotective effect relies on (a) the amount of Q adsorbed on their surface, (b) the amount of Q delivered into the cell, or (c) the effect of MSNs themselves. In the latter case, it is essential to highlight the T-MSN, since thiol-antioxidants contain a nucleophilic thiol group (R-SH) that can directly interact with the electrophilic centers of oxidizing radicals [64]. In this regard, T-MSNs were used to treat bone tissue regeneration, demonstrating antioxidant activity, neutralizing ROS generated in murine osteoblast precursor cells (MC3T3), and protecting against ROS-induced cell damage [61]. However, the mechanism explaining the cytoprotective effect of T-MSN in the present results remains unclear.

**Figure 7:**
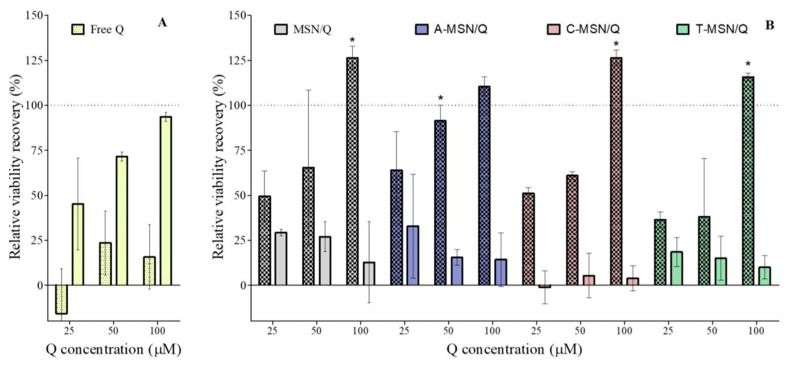
Relative viability recovery of Caco-2 cells exposed to H_2_O_2_ after a pre-incubation with (**A**) free Q for 2 h (pattern) or 18 h (clear) or (**B**) F-MSN/Q (pattern) or its release medium (clear) for 18 h. Data shown are the means ± SD (*n*= 3). Treatments of particles different from their respective concentrations of free Q (18 h) are marked with * (*p* < 0.05).

Figure 7A compares the effects of free or loaded Q on the relative viability recovery of Caco-2 cells. Unlike free Q, all treatments with 100 µM Q-loaded MSNs showed a recovery in relative viability above 100% (Figure 7B). It must be noted that, compared with free Q, this effect was reached without the use of DMSO as a vehicle. Further investigations are needed to clarify the mechanism underlying this observation; nevertheless, the findings presented here suggest that MSNs can protect Q from degradation in cell culture media and prevent Q from forming complexes with media components. In addition, this leads to better delivery of the Q into the cell. All loaded particles showed stronger cytoprotective effects than free Q; however, the mechanisms appear to differ among them.

This work provides insight into the influence of mesoporous silica nanoparticles’ surface chemistry on their interactions with intestinal cells and on their performance in cell culture when used as carriers for quercetin. This research also provides information on the mechanism of quercetin’s effects and the improvement observed when using this nanomaterial. Ultimately, these findings may have future relevance in the study of mesoporous silica nanoparticles as an oral delivery system for polyphenols.

## 4. Conclusions

Mesoporous silica nanoparticles, as well as their aminated, carboxylated, or thiolated modifications, have been demonstrated to be suitable vehicles to preserve and even enhance Q bioactivity. The functional groups added provided models for attractive, repulsive, and neutral interactions between quercetin and each mesoporous material. These modifications altered the release kinetics of Q in cell culture and its cell uptake. In brief, A-MSN exhibited stronger interactions with Q, C-MSN increased Q-cellular uptake, and T-MSN likely showed antioxidant activity. The cytoprotective effect of loaded particles was not due to Q release, which is an innovative finding of this study. Further investigation is needed to elucidate the mechanism underlying the cytoprotective effect of Q-loaded MSNs, which may be attributed to the amount of Q adsorbed on their surface, the amount delivered into the cell, or the effect of MSNs themselves.

## Figures and Tables

**Table 1:** Loading capacity with Q (g quercetin/g particles) and ζ potential of MSN, A-MSN, C-MSN, and T-MSN suspended in MEM.

	Loading Capacity (%)	ζ Potential (mV)
MSN	2.79 ^b^ ± 0.20	−36.89 ^ab^ ± 1.35
A-MSN	5.26 ^a^ ± 0.06	−42.61 ^ab^ ± 1.48
C-MSN	1.85 ^c^ ± 0.23	−44.67 ^b^ ± 1.51
T-MSN	1.26 ^c^ ± 0.34	−36.72 ^a^ ± 2.89

Means (*n* = 3) not connected by the same letter are statistically different (*p* < 0.05).

## Data Availability

The original contributions presented in this study are included in the article. Further inquiries can be directed to the corresponding author.

## Abbreviations

The following abbreviations are used in this manuscript:

Q	Quercetin
MSN	Mesoporous silica nanoparticles
A-MSN	Amine-functionalized mesoporous silica nanoparticles
C-MSN	Carboxyl-functionalized mesoporous silica nanoparticles
T-MSN	Thiol-functionalized mesoporous silica nanoparticles
A-MSN/Q	Amine-functionalized mesoporous silica nanoparticles loaded with quercetin
C-MSN/Q	Carboxyl-functionalized mesoporous silica nanoparticles loaded with quercetin
T-MSN/Q	Thiol-functionalized mesoporous silica nanoparticles loaded with quercetin
